# Diagnostic Yield of High-Resolution Vessel Wall Magnetic Resonance Imaging in the Evaluation of Young Stroke Patients

**DOI:** 10.3390/jcm13010189

**Published:** 2023-12-29

**Authors:** Naaem Simaan, Tamer Jubeh, Fatma Shalabi, Hamza Jubran, Issa Metanis, Yoav Parag, Yoel Schwartzman, Jad Magadlla, John. M. Gomori, Karine Wiegler Beiruti, Jose E. Cohen, Ronen Leker

**Affiliations:** 1 Department of Neurology, Hadassah-Hebrew University Medical Center, Jerusalem 9112102, Israel; naaems@ziv.gov.il (N.S.); fatma.shalabi@gmail.com (F.S.); hamzeh-89@hotmail.com (H.J.); issa.meta@yahoo.com (I.M.); yoelschwartzman@gmail.com (Y.S.); j.magadle@gmail.com (J.M.); 2 Azrieli Faculty of Medicine, Bar Ilan University, Safed 1311502, Israel; 3 Department of Neurology, Ziv Medical Center, Safed 1311001, Israel; tamer0001@hotmail.com; 4 Department of Radiology, Hadassah-Hebrew University Medical Center, Jerusalem 9112102, Israel; pyoav@hadassah.org.il (Y.P.); gomori@cc.huji.ac.il (J.M.G.); 5 Research Wing, Ziv Medical Center, Safed 1311001, Israel; karinebeiruti@ziv.gov.il; 6 Department of Neurosurgery, Hadassah-Hebrew University Medical Center, Jerusalem 9112102, Israel; jcohenns@yahoo.com

**Keywords:** vessel wall, MRI, stroke, young patients

## Abstract

(1) Background: The mechanism responsible for stroke in patients younger than 50 often remains unknown. This study was designed to assess whether high-resolution intracranial vessel wall MR imaging (icVWI) may be instrumental in determining stroke cause. (2) Methods: Young stroke patients with and without an identified cause of stroke despite an exhaustive investigation were prospectively included. Patients who underwent icVWI were compared to those who did not. We next compared patients with and without intracranial vulnerable plaques on icVWI. (3) Results: Overall, 47 young stroke patients were identified over the span of 2 years and included in this study. Of those, 20 (42%) underwent intracranial icVWI. Cancer prevalence was higher among patients who did not have an icVWI study (19% vs. 0% *p* = 0.042) but there were no other significant differences between patients who had an icVWI study and those who did not have an icVWI. Among patients who had an icVWI, 11 (55%) had vulnerable plaques and the remaining nine studies were negative. Patients with positive icVWI scans had significantly higher stroke severity at admission (mean ± SD NIHSS score 5.5 ± 3.5 vs. 1.7 ± 2.3, *p* = 0.012). Patients with positive icVWI scans were more often treated with antiplatelets upon discharge (100% vs. 67%, *p* = 0.038). (4) Conclusions: icVWI can add significant information relevant to stroke pathogenesis and secondary prevention among young stroke patients with a negative exhaustive diagnostic workup.

## 1. Introduction

Ischemic stroke occurring in patients younger than 50 years is not rare and comprises up to 15% of all strokes with incidence rates rising over the last few years [1,2,3,4,5,6,7]. These strokes have high recurrence rates [8] and often do not have benign outcomes [2,3,9]. Stroke in young adults can be secondary to cardioembolism or atherosclerosis but also to uncommon causes at other age groups, such as arterial dissection, use of illicit drugs, vasculitis, infections, and various hypercoagulability syndromes among others [4,6,7,10,11]. Stroke in young adults can also be secondary to isolated intracranial atherosclerotic plaques but the attributes and outcomes of these patients remain largely unexplored [12,13,14]. High-resolution magnetic imaging of the intracranial vessel walls (icVWI) highlighting plaque vulnerability may be a viable tool in the evaluation of the stroke mechanism [15,16,17]. Therefore, the aim of our study was to determine the role of icVWI in the evaluation of the intracranial arteries in young stroke patients.

## 2. Materials and Methods

### 2.1. Study Design and Source of Data

Stroke patients younger than 50 were identified from a cohort of consecutive stroke patients admitted over 2 years to the stroke unit at a large academic center. The study was approved by the institutional ethical committee with an exemption from obtaining informed consent because of the retrospective design and use of anonymized data collection. 

For the current study, we included patients younger than 50 years old with a non-lacunar stroke on imaging. All patients underwent a thorough workup including non-contrast brain computerized tomography (NCCT), CT angiography and perfusion (CTA, CTP), echocardiography with bubble injection, in-patient cardiac monitoring, outpatient Holter monitoring, and laboratory tests for coagulopathies, infections, and occult malignancies. Urine testing for illicit drugs including cannabinoids, cocaine, and amphetamines was also performed on all the patients. Most patients also underwent diffusion weighted (DW) and fluid attenuated inversion recovery (FLAIR) magnetic resonance imaging (MRI) to ascertain the location of the culprit stroke during admission, young stroke patients with negative results from the above-mentioned workup in whom the mechanism responsible for stroke remained unknown were included in the study. Patients with intracranial vessel stenosis larger than 50% were excluded. We specifically used this cutoff because it was used in previous studies [18,19].

Patients with an unidentified stroke cause at the completion of this diagnostic underwent icVWI of the intracranial vessel walls. Imaging was performed on a 3-Tesla Ingenia Scanner (Philips Healthcare, Best, The Netherlands) as detailed elsewhere [19]. All studies were initially completed without contrast followed by contrast-enhanced 3D black blood sequence [19]. icVWI was obtained on axial, sagittal, and coronal planes, and plaque enhancement was considered positive only if it appeared to be localized to the same locations on the vessel wall on all projections [19]. Plaque vulnerability was diagnosed only when enhancement was seen on icVWI [20]. The distribution of the enhancement was considered concentric or eccentric based on imaging. The following data were also gathered: the presence of vessel wall remodeling, plaque hemorrhage, and length and % luminal stenosis [19]. 

Young stroke patients with icVWI-positive scans were compared to young stroke patients who were icVWI-negative. Data collected included demographics, risk factor profile, stroke severity, imaging findings, treatments given, and outcomes. Patients were classified as having favorable outcomes if the modified Rankin score at 90 days post-stroke was equal to or lower than 2. Data on the occurrence of hemorrhagic transformations, mortality, and recurrent stroke rates at 90 days and 1 year were also collected. 

### 2.2. Statistical Analysis

Statistical analysis was performed using the SPSS 29 software (IBM, Omaha, NE, USA). A *p* value of <0.05 was considered significant. The *x*^2^ test or Fisher exact test was used to explore the link between qualitative variables. The student’s *t*-test was used to compare continuous parametric variables and the Mann–Whitney and Median tests were used for nonparametric testing. Multivariable logistic regression models were used to test the effects of different variables on the likelihood of having positive icVWI scans using those variables that yielded *p* values of <0.2 on univariate analyses.

## 3. Results

Overall, 47 young stroke patients who fulfilled the entry criteria were included in this study. Among them, 20 (42%) underwent icVWI. These patients were compared to the 27 (58%) patients who did not undergo icVWI. It was revealed that a diagnosis of concomitant cancer was significantly more common in the group that did not have an icVWI study (19% vs. 0%, *p* = 0.042). However, no statistically significant differences were observed between groups concerning age, sex, and other potential risk factors for stroke including ischemic heart disease, smoking, hyperlipidemia, hypertension, obesity, diabetes, and chronic renal failure. Furthermore, no significant differences were found in stroke severity at admission and discharge in intensive care unit (ICU) admissions. Patients who underwent icVWI were more likely to be treated with antiplatelets (85% vs. 37%, *p* < 0.001) at discharge, while those who did not undergo icVWI were more often treated with an anticoagulant (63% vs. 15%, *p* < 0.001). Favorable outcome rates at discharge and after 90 days, as well as mortality rates, did not differ between the groups (Table 1). 

**Figure 1:**
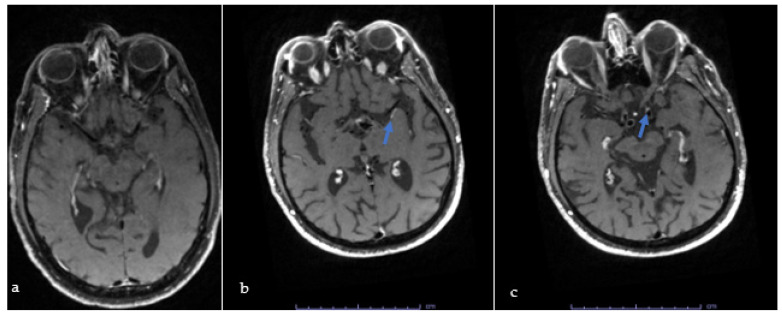
Panel (**a**) shows negative study and panels (**b**,**c**) show positive scans. Blue arrows point to eccentric vessel wall enhancements in the left middle cerebral artery (**b**) and the terminal internal carotid artery (**c**).

More than half of the 20 patients who underwent icVWI, (11, 55%) presented a focal atheroma with plaque enhancement. These patients were compared to the nine patients with negative icVWI studies (Table 2). Patients with positive icVWI had significantly higher stroke severity at admission (5.5% vs. 1.7%, *p* = 0.012) However, age, and other potential risk factors for stroke including hypertension, hyperlipidemia, diabetes, and smoking did not significantly differ between groups. Furthermore, no significant differences were found in treatment with t-PA or thrombectomy. Furthermore, patients who had a positive icVWI study were more likely to be discharged on antiplatelets, whereas those with negative icVWI studies were more likely to be treated with anticoagulants (100% vs. 67% and 33% vs. 0%, respectively, *p* = 0.038). Figure 1 shows icVWI imaging in young stroke patients with stroke. 

## 4. Discussion 

This present study demonstrates that among young stroke patients who remained without an identifiable cause of stroke, despite very thorough investigations, a diagnosis of intracranial atherosclerosis with vulnerable non-stenotic plaque could be the likely cause of stroke in up to 58%. This led to a subsequent change in the strategy of secondary stroke prevention as more patients with evidence of vulnerable intracranial atherosclerotic plaques were discharged on prolonged dual antiplatelet therapy compared to those with negative studies. Additionally, the implementation of strict risk factor control including targeted strategy in lipid-lowering therapy, regular exercise, and more stringent glycemic control may further decrease recurrent stroke risks in these patients [18,21,22]. The results indicate that this strategy could be effective because the rates of stroke recurrence in patients with positive icVWI were similar to those seen in patients with negative icVWI whereas the expected rates should have been higher. 

Whereas the percentage of patients with a positive icVWI may seem high, we assume that these data may reflect the true incidence of intracranial occult non-occlusive atherosclerosis in our population. A presumed cause of stroke could be identified in most patients with a non-lacunar stroke that is not due to steno-occlusive atherosclerotic disease. Common causes for these strokes with an initially undetermined etiology include previously unknown atrial fibrillation, coagulopathies, vasculitis occult malignancy, and patent foramen ovale among others [19]. Prior studies concluded that up to 25% of young stroke patients have an undetermined stroke cause. A recently published study determined that nearly 10% of young stroke patients in France have intracranial stenosis and that most of these cases are secondary to focal intracranial atherosclerosis [14]. However, that study included patients with >50% intracranial stenosis whereas we excluded patients with significant intracranial stenosis and only included patients with <50% intracranial stenosis. In fact, our strict inclusion criteria that included only stroke patients who had a negative extensive workup only left a small proportion of patients without any identifiable stroke cause, which may explain the high likelihood of identifying a potential culprit lesion on icVWI in the current study. 

This study ascertains the importance of high-resolution icVWI in the evaluation of young stroke patients. This advanced technique allows direct intracranial vessel wall characterization and may improve patient management and outcomes. It offers significant information concerning stroke pathogenesis, enhances diagnostic accuracy, and may therefore guide therapeutic decision-making in everyday clinical practice in the future.

It is important to highlight that whereas extracranial carotid non-occlusive plaques have been advocated as the cause of artery-to-artery embolization in a subgroup of patients with cryptogenic stroke [15,23,24,25], the role of intracerebral vulnerable plaques was less often investigated in these patients [16,17] and was only rarely specifically studied previously in young stroke patients. One previous study that explored the role of icVWI in young stroke patients focused on plaque attributes [13] and another study focused on changes in diagnosis after implementation of icVWI to the diagnostic protocol [12]. In agreement with the current results, the latter study found that the diagnosis changed from stroke of unknown cause to atherosclerotic large vessel disease in 39% of 253 young stroke patients [12]. However, the percent stenosis on MR or CT angiography was considerably larger than in the current study, and similarly to Munio et al. [14], they also included patients with >50% stenosis. The novelty of our results lies in showing that the changes in the proposed mechanism responsible for stroke could trigger different secondary prevention treatment strategies resulting in a shift from anticoagulants to antiplatelets. Whether or not this shift proves effective in stroke prevention remains to be seen in larger prospective studies. 

Previous studies have demonstrated that vessel remodeling was associated with ipsilateral stroke and that the prevalence of vulnerable plaques was significantly higher ipsilateral to the stroke [17]. Because clear clinical correlations have been documented before between vessel wall enhancement on icVWI and stroke mechanism [12,13,15,16,26,27,28,29,30,31,32,33,34,35,36], as well as stroke recurrence [20], we specifically choose to use vessel wall enhancement for the assessment of plaque vulnerability. Other markers of plaque instability such as vessel wall thickening and remodeling and the presence of intra-plaque hemorrhage [15,31,37] were less frequently present in young stroke patients with positive icVWI contrast enhancement. Similar to vessel wall enhancement, these markers were non-existent in patients without vessel wall enhancement on icVWI ascertaining their specificity. However, these specific markers appear to be less sensitive than contrast enhancement of the plaques.

Our study is not without limitations. Only selected young stroke patients underwent icVWI. However, they did not differ in any vascular risk factor compared to patients who did not undergo icVWI, which suggests that the results are generalizable to all young stroke patients with a negative workup. Nevertheless, we cannot completely exclude the possibility that some young stroke patients with strokes presumed to be secondary to other competing mechanisms, such as atrial fibrillation, might also have vulnerable intracranial plaques. Therefore, we suggest that future prospective studies should perform icVWI as part of the standard workup in young stroke patients. In addition, the retrospective design of this study is subject to numerous biases. Nevertheless, the data collected prospectively represent real-life stroke care. Therefore, we believe that our data can serve as an initial hypothesis-generating study. Since extracranial VWI was not performed in any of the patients, it is not possible to exclude that, at least in some cases, extracranial and intracranial plaques co-existed and that some patients with negative icVWI studies may actually have vulnerable non-stenotic extracranial plaque. However, routine CTA and MRA studies did not demonstrate significant (>50%) extracranial stenosis in any of the included patients. Finally, the small sample size could limit the accuracy and generalizability of our findings suggesting the importance of future studies with a larger sample size to confirm our findings.

## 5. Conclusions

This study puts forward icVWI as a valuable tool for the determination of the possible pathogenesis of stroke in young stroke patients. This information could be important for planning secondary prevention strategies in this group of patients. Further prospective studies testing the yield of icVWI in young stroke patients are needed to confirm our findings.

## Figures and Tables

**Table 1:** Comparison between young stroke patients according to icVWI completion status.

Variable/Group	icVWI Done (*n* = 20)	icVWI Not Done (*n* = 27)	*p* Value
Age (mean, SD)	43.3 (11)	46.2 (6.7)	0.273
Gender male (%)	13 (65)	14 (52)	0.579
Hypertension (%)	4 (20)	8 (30)	0.454
Diabetes (%)	4 (20)	7 (26)	0.635
Hypercholesterolemia (%)	3 (15)	8 (30)	0.242
Smoking (%)	9 (45)	10 (37)	0.582
Ischemic heart disease (%)	0 (0)	3 (11)	0.123
Prior stroke (%)	2 (10)	2 (7)	0.753
Cancer (%)	0 (0)	5 (19)	0.042
Tissue plasminogen activator (%)	2 (10)	6 (22)	0.270
Endovascular Thrombectomy (%)	6 (30)	4 (15)	0.315
Discharge medications (%)			0.001
Antiplatelet	17 (85)	10 (37)	0.001
Anticoagulation	3 (15)	17 (63)	0.001
Admission NIHSS (mean, SD)	3.8 (3.6)	5.2 (5.6)	0.315
Discharge NIHSS (mean, SD)	3.2 (4.4)	4.2 (8.4)	0.652
NIHSS at 3 months (mean, SD)	2 (3.2)	2.2 (2.1)	0.902
mRS 0–2 prior to admission (%)	16 (80)	26 (96)	0.073
mRS 0–2 upon discharge (%)	14 (70)	23 (85)	0.209
mRS 0–2 at 3 months (%)	17 (85)	23 (85)	0.986
ICU (%)	7 (35)	8(30)	0.696
Mortality (%)	0 (0)	3 (11)	0.123

icVWI—intracranial vessel walls imaging, NIHSS—National Institutes of Health Stroke Scale, mRS—modified Rankin score, ICU—intensive care admission.

**Table 2:** Characteristics of patients with positive and negative icVWI scans.

Variable/Group	VWI+ (*n* = 11)	VWI− (*n* = 9)	*p* Value
Age (mean, SD)	43.7 (9.9)	42.8 (12.8)	0.854
Hypertension (%)	2 (18)	2 (22)	0.822
Diabetes (%)	2 (18)	2 (22)	0.822
Hypercholesterolemia (%)	2 (18)	1 (11)	0.660
Smoking (%)	7 (64)	2 (22)	0.064
Prior stroke (%)	2 (18)	0 (0)	0.178
Tissue plasminogen activator (%)	1 (9)	1 (11)	0.881
Endovascular thrombectomy (%)	4 (36)	2 (22)	0.269
Discharge medications (%)			0.038
Dual Antiplatelet	11 (100)	6 (67)	
Anticoagulation	0 (0)	3 (33)	
Admission NIHSS (mean, SD)	5.5 (3.5)	1.7 (2.3)	0.0120
Discharge NIHSS (mean, SD)	3.7 (4.6)	2.6 (4.5)	0.575
mRS 0–2 prior to admission (%)	9 (81)	7 (78)	0.012
mRS 0–2 upon discharge (%)	7 (64)	7 (78)	0.575
mRS 0–2 at 3 months (%)	8 (73)	9 (100)	0.321
Recurrent stroke (%)	4 (36)	1 (11)	0.243
Intra-plaque hemorrhage (%)	1 (11)	0 (0)	0.331
Wall remodeling (%)	4 (44)	0 (0)	0.031
Plaque length (mean, SD)	2.5 (2.2)	0 (0)	0.008
Stenosis percent (mean, SD)	33.3 (33.5)	0 (0)	0.013
ICU (%)	6 (55)	1 (11)	0.043

icVWI—intracranial vessel wall imaging, NIHSS—National Institutes of Health Stroke Scale, mRS—modified Rankin score, ICU—intensive care admission.

## Data Availability

Full data are available following a formal request and in compliance with state regulations.
